# 539. Efficacy in Multiple SARS-CoV-2 Animal Models Supports Phase 3 Dose Selection for Obeldesivir

**DOI:** 10.1093/ofid/ofad500.608

**Published:** 2023-11-27

**Authors:** Jared Pitts, Darius Babusis, Rita Humeniuk, David Martinez, Robert M Cox, Alexandra Schäfer, Nicholas C Riola, Joy Feng, Venice Du Pont, Olena Anoshchenko, Meghan Vermillion, Mazin Abdelghany, Robert H Hyland, Sandhya Girish, Timothy P Sheahan, Richard K Plemper, Ralph S Baric, Tomas Cihlar, Joe Llewellyn, Helen Winter, Roy Bannister, Raju Subramanian, Richard L Mackman, John P Bilello

**Affiliations:** Gilead Sciences, Inc., Foster City, California; Gilead Sciences, Inc., Foster City, California; Gilead Sciences, Inc., Foster City, California; University of North Carolina, Chapel Hill, North Carolina; Institute for Biomedical Sciences, Georgia State University, Atlanta, Georgia; University of North Carolina, Chapel Hill, North Carolina; Gilead Sciences, Inc., Foster City, California; Gilead Sciences, Inc., Foster City, California; Gilead Sciences, Inc., Foster City, California; Gilead Sciences, Inc., Foster City, California; Lovelace Biomedical Research Institute, Albuquerque, NewMexico; Gilead Sciences, Inc., Foster City, California; Gilead Sciences, Inc., Foster City, California; Gilead Sciences, Inc., Foster City, California; University of North Carolina, Chapel Hill, North Carolina; Institute for Biomedical Sciences, Georgia State University, Atlanta, Georgia; Institute for Biomedical Sciences, Georgia State University, Atlanta, Georgia; Gilead Sciences, Inc., Foster City, California; Gilead Sciences, Inc., Foster City, California; Gilead Sciences, Inc., Foster City, California; Gilead Sciences, Inc., Foster City, California; Gilead Sciences, Inc., Foster City, California; Gilead Sciences, Inc., Foster City, California; Gilead Sciences, Inc., Foster City, California

## Abstract

**Background:**

Remdesivir (RDV, Veklury) is the first FDA-approved direct-acting antiviral treatment for COVID-19. While RDV requires IV administration, obeldesivir (ODV, GS-5245) is an oral prodrug of GS-441524, the parent nucleoside of RDV, designed for effective oral delivery. ODV is being tested in two Phase 3 clinical studies for outpatient treatment of COVID-19.

**Methods:**

In vitro ODV activity against SARS-CoV-2 was assessed in A549-hACE2 cells. In vivo therapeutic efficacy of oral ODV was evaluated in mouse, ferret, and African Green Monkey (AGM) SARS-CoV-2 models. GS-441524 pharmacokinetics (PK) following ODV oral administration was assessed in animals and in a Phase 1 study in humans.

**Results:**

ODV is a potent inhibitor of SARS-CoV-2 in vitro (EC_50_ = 1.9 µM) and showed 2- to 7-fold increased GS-441524 oral bioavailability in multiple animal models compared to parent GS-441524. Oral ODV reduced infectious lung viral loads by 2.8 log_10_ plaque forming units (pfu)/g tissue and ameliorated viral pathophysiological effects in a pathogenic mouse model of SARS-CoV-2 when dosed at 10 mg/kg twice daily beginning 12 hours post infection (hpi). In a SARS-CoV-2 asymptomatic upper airway infection ferret model, oral ODV dosed once daily at 20 mg/kg starting 12 hpi reduced nasal infectious viral titers by >3.3 log_10_ pfu/mL. Viral transmission among co-housed ferrets was also prevented by ODV. Once daily oral administration of 60 mg/kg ODV in SARS-CoV-2 infected AGMs initiated 8 hpi significantly reduced viral loads in bronchoalveolar lavage and multiple respiratory tissues. Efficacious oral doses of ODV in the mouse, ferret and AGM models were achieved at estimated plasma GS-441524 exposures (AUC_0-24h_) of 36, 98 and 111 µM·h, respectively. Based on PK data from a Phase 1 dose escalation study in healthy volunteers, an ODV dose of 350 mg twice daily (BID) is expected to achieve plasma GS-441524 exposures (77-103 µM·h) in the efficacious range derived from these animal models.

**Conclusion:**

ODV is a promising investigational oral COVID-19 treatment based on its preclinical in vitro and in vivo SARS-CoV-2 efficacy, its oral bioavailability in preclinical species, and its oral PK and safety profile in a Phase 1 trial. Collectively, these data support the 350 mg BID dose for the ongoing Phase 3 clinical trials.

**Disclosures:**

**Jared Pitts, PhD**, Gilead Sciences, Inc.: Employee|Gilead Sciences, Inc.: Stocks/Bonds **Darius Babusis, BA**, Gilead Sciences, Inc.: Employee|Gilead Sciences, Inc.: Stocks/Bonds **Rita Humeniuk, PhD**, Gilead Sciences, Inc.: Employee|Gilead Sciences, Inc.: Stocks/Bonds **Nicholas C. Riola, BS**, Gilead Sciences, Inc.: Employee|Gilead Sciences, Inc.: Stocks/Bonds **Joy Feng, PhD**, Gilead Sciences, Inc.: Employee|Gilead Sciences, Inc.: Stocks/Bonds **Venice Du Pont, PhD**, Gilead Sciences, Inc.: Employee|Gilead Sciences, Inc.: Stocks/Bonds **Olena Anoshchenko, PhD**, Gilead Sciences, Inc.: Employee|Gilead Sciences, Inc.: Stocks/Bonds **Meghan Vermillion, DVM, PhD**, Gilead Sciences, Inc.: Employee **Mazin Abdelghany, MD**, Gilead Sciences, Inc.: Employee|Gilead Sciences, Inc.: Stocks/Bonds **Robert H. Hyland, DPhil**, Gilead Sciences, Inc.: Employee|Gilead Sciences, Inc.: Stocks/Bonds **Sandhya Girish, PhD**, Gilead Sciences, Inc.: Employee|Gilead Sciences, Inc.: Stocks/Bonds **Tomas Cihlar, PhD**, Gilead Sciences, Inc.: Employee|Gilead Sciences, Inc.: Stocks/Bonds **Joe Llewellyn, PharmD**, Gilead Sciences, Inc.: Employee|Gilead Sciences, Inc.: Stocks/Bonds **Helen Winter, PhD**, Gilead Sciences, Inc.: Employee|Gilead Sciences, Inc.: Stocks/Bonds **Roy Bannister, PhD**, Gilead Sciences, Inc.: Employee|Gilead Sciences, Inc.: Stocks/Bonds **Raju Subramanian, PhD**, Gilead Sciences, Inc.: Employee|Gilead Sciences, Inc.: Stocks/Bonds **Richard L. Mackman, PhD**, Gilead Sciences, Inc.: Employee|Gilead Sciences, Inc.: Stocks/Bonds **John P. Bilello, PhD**, Gilead Sciences, Inc.: Employee|Gilead Sciences, Inc.: Stocks/Bonds

